# Excitatory/Inhibitory balance in autism spectrum disorders: Integrating genetic, neurotransmitter and computational perspectives

**DOI:** 10.3934/Neuroscience.2025031

**Published:** 2025-12-11

**Authors:** Dilip Madia, Mujibullah Sheikh, Anil Pethe, Darshan Telange, Surendra Agrawal

**Affiliations:** Datta Meghe College of Pharmacy, (DMIHER), Deemed to be University, Sawangi (Meghe), Wardha 442001, India

**Keywords:** autism spectrum disorder, E/I balance, computational modeling, GABA, glutamate

## Abstract

Computational modeling of excitatory/inhibitory (E/I) balance offers transformative insights into the neurobiological underpinnings of autism spectrum disorder (ASD). In this review, we examined the integration of neurotransmitter dynamics and genetic factors into multiscale computational frameworks to elucidate the mechanisms driving E/I dysregulation in ASD. We explored the pivotal roles of glutamate and GABA, the primary excitatory and inhibitory neurotransmitters, and the modulatory impact of serotonin and dopamine (DA), in shaping neural circuit stability, behavioral outcomes, and ASD core symptoms. Genetic mutations affecting synaptic proteins such as SHANK3, GRIN2A, and GABRB3 were highlighted for their capacity to disturb synaptic scaffolding and glutamatergic and GABAergic signaling, thereby shifting the E/I ratio. Computational approaches, ranging from detailed neuronal simulations to neural mass and spiking network models, captured the heterogeneous manifestations of E/I imbalance and aligned with molecular, neuroimaging, and electrophysiological findings in ASD. We discussed how these models informed individualized diagnostic strategies, enabled prediction of treatment responses, and offered targets for precision medicine. Major challenges included methodological inconsistencies, neurochemical measurement discrepancies, polygenic interactions, and the translation of model predictions into clinical practice. We concluded that the integration of neurotransmitter and genetic data within advanced computational models represents a significant advance toward unraveling ASD pathophysiology, with the promise of developing dynamic, personalized interventions. Ongoing efforts should emphasize longitudinal data, multiomic integration, sex-specific trajectories, and cross-disciplinary collaboration to further the clinical applicability and translational potential of computational E/I balance modeling in autism research.

## 1. Introduction

A balanced excitatory/inhibitory rate is essential for maintaining neuronal stability and ensuring proper brain function. The E/I balance is primarily intermediated by natural excitability, which is governed by an array of voltage-gated ion channels, and extrinsic excitability, which is maintained through a counterbalance between excitatory and inhibitory synaptic transmission [1]. By the 1950s, electrophysiological studies by Eccles established gamma-aminobutyric acid (GABA) and glutamate as central inhibitory/excitory neurotransmitters, linking E/I dynamics to synaptic malleability. This framework evolved through Hebbian principles, emphasizing that neural stability requires precise tuning between excitatory pyramidal neurons (driven by glutamatergic synapses) and inhibitory interneurons (GABAergic systems) [2]. Disruptions to this equilibrium, whether due to inherited mutations, synaptic dysfunction, or network-level differences, alter neural coding and lead to abnormal oscillations and impaired information processing [3].

Computational modeling has become a valuable tool for studying E/I balance. Early models, such as the Hodgkin–Huxley model, detail action potentials. Computational models are being utilized to investigate E/I dynamics, simulate neuronal population dynamics, and illustrate the effects of changes in synaptic weights, neurotransmitter levels, or receptor kinetics that influence neural oscillations. The integration of computational models with experimental data enhances the understanding of how the E/I balance shapes circuit functioning and its dysregulation in disorders such as ASD [4].

**Figure 1.**
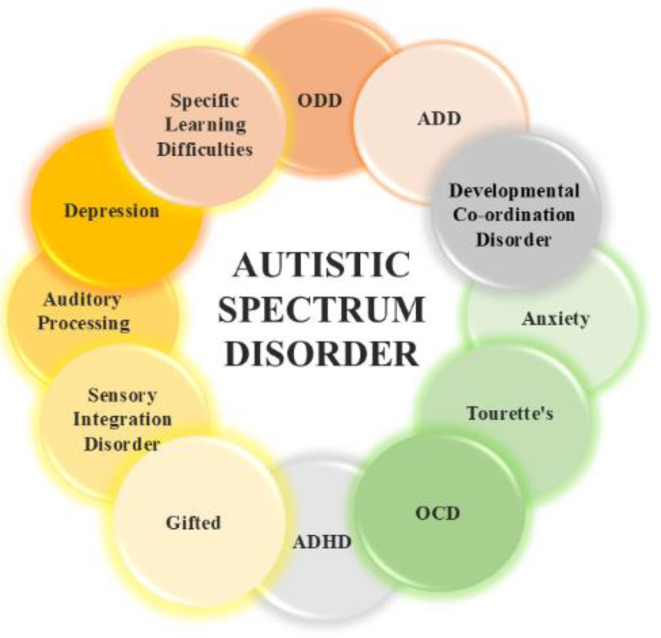
Common comorbidities of ASD, highlighting its neurodevelopmental, psychiatric, and cognitive heterogeneity.

E/I imbalance has been suggested as a network-level theory for neurological and behavioral dysfunction in individuals with neurodevelopmental disorders, including ASD [5]. ASD is defined by disability in social interaction and communication, including repetitive activities [6]. Research has indicated that a change in the E/I balance in individuals with ASD could result in abnormal size-dependent modulation of motion perception [7]. Some researchers have suggested that subjects with EEG abnormalities exhibit different physiological subgroups within ASD, with epileptiform and nonepileptiform EEG abnormalities corresponding to contrasting E/I balance disruptions [8]. The complex interplay of risk genes and underlying neurobiological mechanisms is further evidenced by the frequent co-occurrence of ASD with a range of other neurological and developmental conditions, as visually summarized in Figure 1. These common comorbidities include challenges such as oppositional defiant disorder (ODD), attention deficit hyperactivity disorder (ADHD), depression, anxiety, OCD, Tourette's, and specific learning difficulties. Additionally, sensory processing and integration disorders, developmental coordination disorders, and giftedness can present alongside ASD, highlighting the broad impact of E/I dysregulation (Figure 1) [9]. The balance between E/I is essential for neural stability and optimal brain function, with E/I dysregulation associated with neurological conditions, including ASD. Historically, research has illustrated the significance of inhibitory processes in mitigating neuronal excitement [4]. Early studies clarified the importance of GABA synapses in modulating cortical activity, whereas later studies established the role of E/I balance in sensory processing, learning, and memory consolidation [5].

Our objective of this review is to explore how integrating neurotransmitter dynamics and genetic factors into computational models can deepen our understanding of the E/I imbalance in ASD. By synthesizing evidence from molecular neurobiology, synaptic physiology, and computational neuroscience, we aim to highlight the potential of multiscale modeling approaches to capture the complex interplay between glutamatergic/GABAergic signaling and ASD-associated genetic mutations. This integrated perspective provides a mechanistic framework for investigating ASD pathophysiology and guiding the development of precision-based diagnostic and therapeutic strategies.

## 2. Materials and methods

A comprehensive literature review was conducted using databases such as PubMed, Web of Science, Scopus, and Google Scholar, with keywords related to ASD, E/I balance, neurotransmitters (GABA, glutamate), genetics, and computational modeling. Inclusion criteria focused on peer-reviewed studies examining E/I balance, neurotransmitter dynamics, genetic factors, and computational simulation in ASD, while excluding non-peer-reviewed and irrelevant studies. Titles and abstracts were screened, followed by full-text assessment to extract data on study design, methodology, sample characteristics, and major findings. Each study was critically appraised for methodological quality and bias. Synthesized data enabled identification of consistent themes and discrepancies, facilitating an integrated understanding of neurotransmitter and genetic influences on E/I balance in ASD. The literature search was performed across four major databases: PubMed, Web of Science, Scopus, and Google Scholar, using keywords such as “ASD”, “E/I balance”, “GABA”, “glutamate”, “genetic factors”, “neurotransmitter dynamics”, and “computational modeling”. The search query combined these terms to capture relevant studies focused on the computational modeling of E/I balance in ASD. Studies were included if they were peer-reviewed and focused on the E/I balance in ASD, neurotransmitter dynamics (particularly GABA and glutamate), genetic factors related to the E/I balance, and computational models simulating this balance. Exclusion criteria consisted of non-peer-reviewed articles, studies unrelated to ASD, or research lacking sufficient methodological detail.

Titles and abstracts were initially screened to identify potential studies, followed by full-text assessment applying the inclusion and exclusion criteria. Key data extracted included experimental design, methodology, sample characteristics (age, sex, diagnostic criteria), and major findings related to neurotransmitter levels, genetic influences, and computational strategies.

Each study was evaluated for methodological rigor and potential bias. A critical analysis of the data and computational models was performed. Synthesized findings highlighted cohesive themes and revealed discrepancies, providing an integrated understanding of how neurotransmitter dynamics and genetic factors influence the E/I balance in ASD.

## 3. Conceptual knowledge

### 3.1. Autism

**Figure 2.**
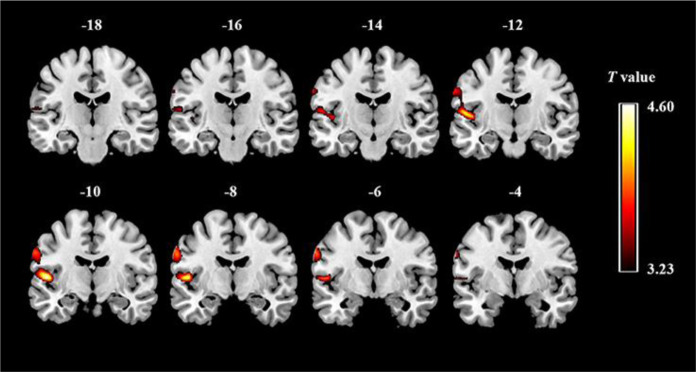
Brain regions with altered gray matter volume distinguishing individuals with ASD from typically developing (TD) individuals. Red highlighted areas represent regions with significantly increased gray matter volume in individuals with ASD compared with TD controls (ASD > TD), indicating that neuroanatomical differences are associated with autism-related brain development. Copyright © 2017 Wang, Fu, Chen, Duan, Guo, Chen, Wu, Xia, Wu and Chen. This is an open-access article distributed under the terms of the Creative Commons Attribution License (CC-BY) [3].

ASD is a heterogeneous neurodevelopmental syndrome characterized by a basic lack of social communication and the existence of restricted, repetitive patterns of behavior, hobbies, or activities [1]. The understanding of autism has developed from early descriptions of isolated features to a spectrum model stressing dimensional presentations and personalized support needs [2]. Neurobiologically, ASD involves abnormal neuronal connections and cortical architecture. This is robustly indicated by structural MRI studies demonstrating gray matter changes that are highly predictive of ASD categorization, achieving significant accuracy (e.g., 95.7% precision in GM-VGG-Net models) [3],[4]. For example, Figure 2 visually illustrates statistically significant differences in gray matter volume, shown as clusters of altered activity using hot colors (yellow and red) that represent higher T values across a series of coronal brain slices. These marked regions indicate loci where gray matter structure differs between groups, providing direct empirical evidence of distinct neuroanatomical signatures in ASD. This figure underscores the structural underpinnings of the disorder, reinforcing the connection between brain morphology and core ASD features.

**Figure 3.**
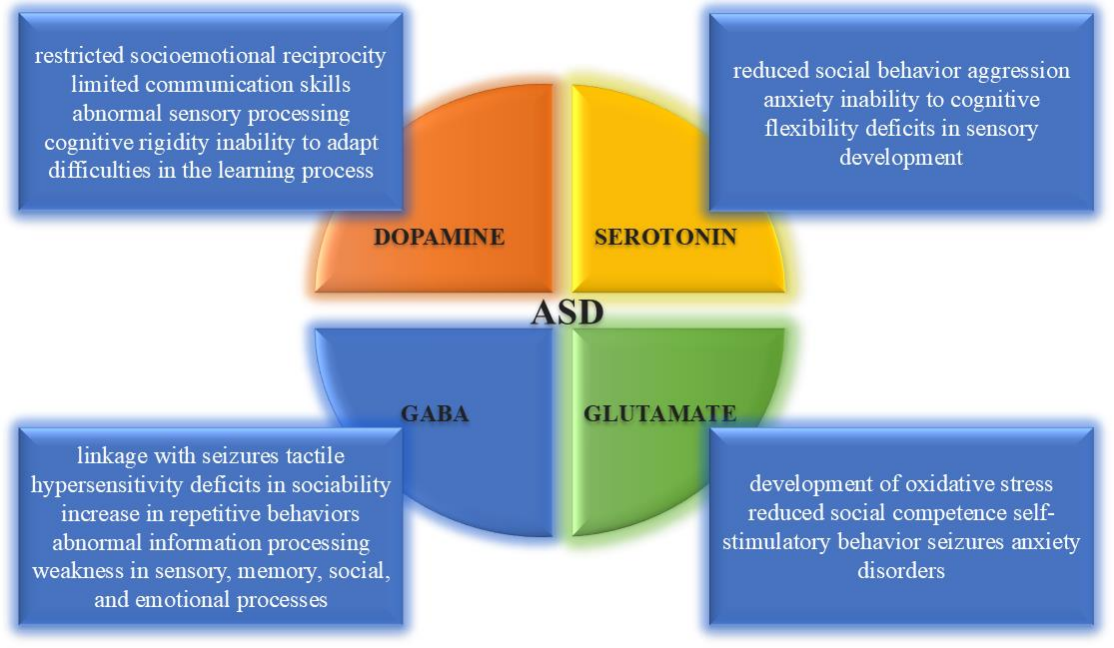
Role of key neurotransmitters in the development of ASD symptoms.

In addition to structural brain changes, neurochemical imbalances are of paramount importance in the symptomatology of ASD. Abnormalities in key neurotransmitters, specifically dopamine (DA), serotonin, GABA, and glutamate, are attributed to the numerous behaviors and cognitive deficiencies observed in individuals with ASD. Figure 3 comprehensively illustrates these associations, highlighting how dysregulation of each neurotransmitter system contributes to distinct symptom clusters. For example, DA imbalances are linked to restricted socioemotional reciprocity and cognitive rigidity, reflecting the neurotransmitter's critical involvement in motivation, learning, and adaptive behavior. Serotonin dysregulation is implicated in reduced social behavior, increased aggression, and deficits in cognitive flexibility, as well as altered anxiety regulation and repetitive behaviors. Furthermore, abnormalities in the GABA system are associated with an increase in repetitive behaviors, disrupted information processing, and a heightened vulnerability to seizures, all due to impaired inhibitory signaling and altered network excitability. Conversely, glutamate dysregulation contributes to the development of oxidative stress, reduced social competence, self-stimulatory behaviors, and anxiety disorders. These neurochemical disruptions collectively underpin the core symptoms of ASD, including social dysfunctions, anxiety, repetitive behaviors, and sensory dysfunctions. The definition and diagnostic criteria of autism have evolved over the years under the impact of research and neurodiversity movements [5]. According to the knowledge enshrined in the DSM-5, attachment importance is given to problems in social interaction, the ability to communicate with others, and the existence of restricted and repetitive behavior [6],[7]. These core symptoms may have various levels of severity, thus resulting in a heterogeneous presentation of individuals with ASD [8]. Early diagnosis can usually be detected by the age of 2, and this situation can be subjected to early therapeutic intervention where long-term intellectual, behavioral and functional problems can be mitigated [9].

The etiology of ASD is multifactorial, i.e., genetic and environmental factors are involved [10],[11]. Several genes are related to this disorder, and the possibility of de novo germline mutations is posited [12]. Moreover, imbalances in the gut microbiome and maternal immune imbalance are considered possible factors [12],[13]. Dysbiosis of the gut, namely, changes in the abundance of Firmicutes, Proteobacteria, Actinomyces, and Bacteroidetes, may result in elevated levels of endotoxins and disorders of metabolites, which may affect brain activity [13]. ASD can also be accompanied by other coexisting conditions, such as anxiety disorders and otolaryngological and sleeping disorders [14]. The prevalence rates of anxiety disorders are very high, and anxiety disorders may have adverse effects on educational, social, and health functions [15]. Otological problems may also interfere with communication and development, including hearing loss, middle ear infection, otitis media, and auditory processing disorders, among others. There are also sleeping disorders, which may affect behavior and thus quality of life [15]. Treatment involves therapies such as cognitive–behavioral therapies, sensory integration therapy, and parent education to provide a state of well-being and better social and cognitive performance [12].

### 3.2. Neurotransmitters

Neurotransmitters are chemicals that are released via synapses by neurons that mediate the synaptic transmission process by either stimulating or preventing the passage of nerve impulses [16]. These substances are stored in vesicles at neuron terminals prior to their release [17]. These compounds are retained in vesicles at the terminals of neurons before their release. The level of neurotransmitters is important for monitoring and treating mental disorders [18]. Neurotransmitters are analyzed via analytical techniques, including brain microdialysis, high-pressure liquid chromatography (HPLC), mass spectrometry (MS), capillary electrophoresis (CE), electroencephalography (EEG), proton nuclear magnetic resonance, and magnetic resonance imaging (MRI) (Table 1) [19],[20].

**Table 1.** Overview of analytical methods for neurotransmitter assessment in the brain.

**Analytical Method**	**Function**	**Ref**
**Brain Microdialysis**	Invasive technique to collect extracellular fluid from specific brain regions for measuring neurotransmitter concentrations	[21]–[23]
**High-Pressure Liquid Chromatography (HPLC)**	Separates and quantifies chemical substances, including neurotransmitters, often from microdialysis samples	[21],[24]
**Mass Spectroscopy (MS)**	Identifies and quantifies neurotransmitters based on mass-to-charge ratio, often coupled with HPLC for greater specificity	[25]
**Capillary Electrophoresis (CE)**	Separates and quantifies neurotransmitters based on charge and size, offering high resolution and sensitivity	[25]
**Electroencephalography (EEG)**	Noninvasive technique that records brain electrical activity, indirectly assessing neurotransmitter function via neural oscillations	[12]
**Proton Nuclear Magnetic Resonance**	Uses magnetic properties of atomic nuclei to quantify neurotransmitters and brain metabolites	[26]
**Magnetic Resonance Imaging (MRI)**	Noninvasive imaging to assess blood oxygenation, flow, and pH, providing structural and functional data related to neurotransmitter activity	[27]

Precision techniques such as nanoparticles and electrochemical sensors make monitoring and detection more specific because of their measured sensitivity, detection thresholds, rapid response, and real-time monitoring capabilities [28],[29]. Functionally, neurotransmitters are broadly classified into three major categories, each of which plays a distinct role in neuronal signaling:

- Excitatory neurotransmitters: These substances, including glutamate (Glu) and DA, primarily excite target cells, increasing the likelihood of an action potential.
- Inhibitory neurotransmitters: Conversely, these neurotransmitters, notably GABA and serotonin (5-HT), inhibit or block the activity of nerve cells, reducing the likelihood of firing.
- Modulatory neurotransmitters: This category, which includes DA and serotonin (5-HT), regulates the activity of other neurotransmitters and can act on multiple cells simultaneously, often over a longer timescale.

**Figure 4.**
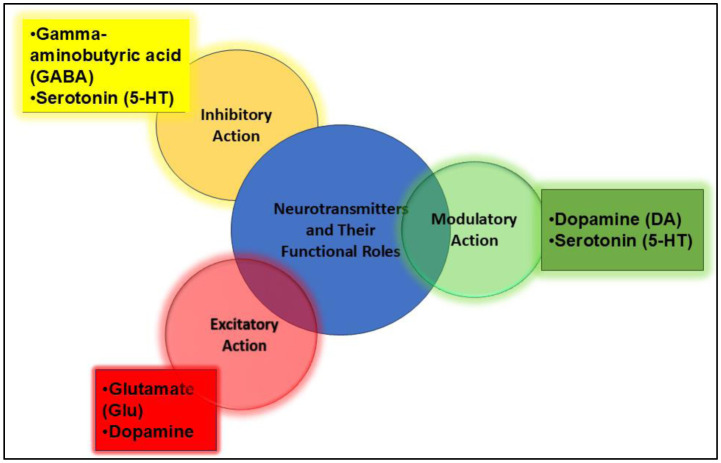
Roles of major neurotransmitters in the central nervous system.

These classifications and their representative neurotransmitters, along with their overlapping functional roles, are visually summarized in Figure 4. The diagram effectively illustrates the multifaceted actions of neurotransmitters; for example, DA and serotonin each display roles that span both modulatory and, respectively, excitatory or inhibitory effects, emphasizing the complexity of neurotransmitter impact on brain function. However, it is noted that, while these conceptual frameworks and classifications are visually represented, we not include figures that directly present simulation results (visualizations of model outputs) or architectural illustrations of neural models relevant to E/I balance disruptions in ASD. Addressing this gap will involve the addition of new figures explicitly depicting model structures and the quantitative outcomes of E/I imbalance simulations, ensuring the manuscript more fully represents theoretical and empirical modeling aspects [30].

**Figure 5.**
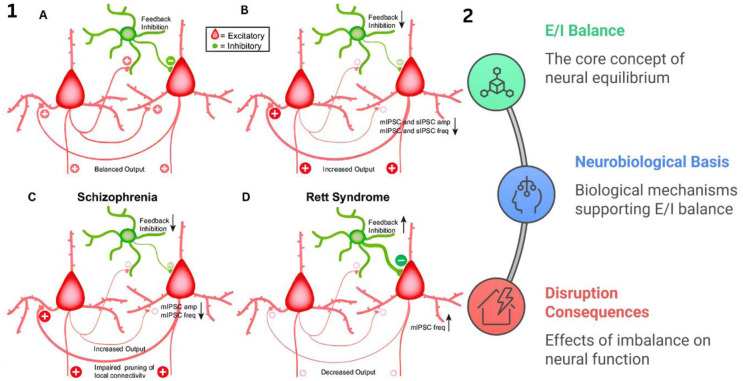
1. This figure demonstrates how shifts in E/I balance drive changes in neural circuit excitability and underlie the pathophysiology of major brain disorders. (A) Healthy neural circuits exhibit an E/I balance between excitatory pyramidal neurons and inhibitory interneurons, resulting in stable output. (B) Animal models of epilepsy show reduced inhibitory synaptic transmission, including decreased amplitude and frequency of miniature (mIPSC) and spontaneous (sIPSC) inhibitory postsynaptic currents, leading to increased circuit excitability. (C) Schizophrenia models display further reductions in feedback inhibition, lower mIPSC amplitude/frequency, and impaired pruning of synaptic connectivity, culminating in heightened excitatory output. (D) In Rett syndrome models, increased inhibitory synaptic activity (mIPSC frequency) and excessive feedback inhibition produce decreased network output. The diagram uses line thickness and plus/minus signs to represent changes in synaptic strength and activity, respectively. This diagram is adapted from reference [35] under the CC-BY 4.0 license. 2. E/I balance is the core principle of neural equilibrium and is sustained by neurobiological mechanisms that adjust synaptic transmission and circuit function. Disruption of this balance alters neural processing and can drive the cognitive, behavioral, and neurological symptoms observed in neurodevelopmental and neuropsychiatric disorders, making E/I balance a key concept in diagnostic and therapeutic strategies.

One type of serotonin (5-HT) is produced in the dorsal raphe nuclei and median raphe nuclei of the caudal midbrain and has an inhibitory role in behaviors such as feeding habits, weight regulation, aggression, obsessive–compulsive disorder (OCD), alcoholism, anxiety, and the regulation of emotions and motors [31]–[33]. It is also involved in the processes of sleep, circadian rhythms, stabilization of breathing, and processing a reward [33]. Serotonin is particularly crucial in neuronal development, cortical plasticity, the morphogenesis of synaptic connections, and the formation of patterned cortical connectivity within the glutamatergic system Figure 5 (1, A) [34]. Figure 5 (2) provides a visual summary of the key regulatory roles of 5-HT, illustrating its broad impact across brain functions and its relationship with other neurotransmitter systems, as well as the fundamental concept of excitation/inhibition (E/I) balance that underpins neural equilibrium and circuit stability. In pathological contexts, such as ASD, serotonin dysregulation is closely linked to core behavioral symptoms and neural circuit disruptions. This is reflected in specific alterations of E/I balance, as conceptualized in panels B, C, and D in Figure 5 (1). Panel B shows increased network output due to reduced feedback inhibition, while panel C depicts schizophrenia-associated impaired synaptic pruning and increased excitation. Panel D illustrates decreased network output characteristic of Rett syndrome, highlighting diverse consequences of E/I imbalance on neural functioning [34].

DADA, a catecholamine, is critical in modulating behavior, mediating reward mechanisms, and influencing social motivation [36]. DA dysregulation is involved in defined neurobehavioral problems in ASD: overactivity in the orbitofrontal–limbic circuit is related to emotional dysregulation, impulsiveness and aggression, whereas prefrontal DA insufficiency is linked to cognitive deficits [37]. Most of the effects of DA are mediated by one or more of the following important pathways: Nigrostriatal, which involves motor control and contributes to Parkinson's disease; mesolimbic, which is associated with reward and emotional aspects; mesocortical, which is associated with cognition and emotion; and tuberoinfundibular, which plays a role in the secretion of prolactin [38],[39].

Glutamate (Glu) is abundant and the most numerous excitatory neurotransmitter in the central nervous system (CNS), accounting for virtually all elements of brain performance. More than two-thirds of synapses in the neocortex and hippocampus use glutamate, so glutamate is the most widespread excitatory neurotransmitter in the mammalian brain [40]. Glutamate facilitates fast synaptic transmission and is crucial for synaptic plasticity, the ability of synapses to strengthen or weaken over time, which underpins learning, memory formation, and adaptive behavior [41]–[43].

Functionally, glutamate is activated by various types of receptors at synaptic junctions, with the best characterized being ionotropic N-methyl-D-aspartate (NMDA), alpha-amino-3-hydroxy-5-methyl-4-isoxazolepropionic acid (AMPA) and kainate receptors and metabotropic glutamate receptors. These subtypes enable glutamate to control ion influx (of calcium) and activate protein kinases and trigger complex signaling cascades that change gene expression and neural activity in the circuit, which are all essential to the encoding and learning processes of memories [44],[45]. Long-term potentiation (LTP), a well-known cellular mechanism for learning and memory, is particularly dependent on NMDA receptor activation by glutamate. Glutamate is produced mainly at the biochemical level through glutamine via a process called the glutamate–glutamine cycle. It is a process of metabolic interaction between neurons and astrocytes, with astrocytes nurturing glutamate into glutamine to subsequently recycle it back to neurons to produce glutamate. This is necessary to replenish neurotransmitter pools and to facilitate glutamatergic communication successfully [46],[47]. The precursor glutamine is derived from dietary sources rich in protein, such as meat, dairy, and eggs, emphasizing the nutritional dependence of neurotransmitter synthesis.

Glutamate acts almost exclusively within the CNS, and its signaling is critical at nearly all excitatory synapses. This widespread presence also makes the CNS particularly sensitive to disruptions in glutamate homeostasis. Excessive accumulation of glutamate in the extracellular space due to impaired uptake or excessive release can lead to excitotoxicity, a harmful process that triggers calcium overload, oxidative stress, and neuronal death. This excitotoxic mechanism is implicated in various neurological disorders, including Alzheimer's disease, stroke, epilepsy, and amyotrophic lateral sclerosis (ALS) [48],[49].

GABA, the main inhibitory neurotransmitter in the brain, is very important because it inhibits the neuronal excitability that balances the system in neural circuits. In addition to its inhibitory actions, GABA plays more fundamental roles in a number of neurodevelopment processes, including cell proliferation, migration, synaptic development, differentiation, and programmed cell death (apoptosis) [50],[51]. Disturbance in GABAergic signaling is also associated with information processing disorders because malfunctioning of the GABAergic system may interfere with synchronizing and modulating the neural network needed by the cognitive process [52]. The loss of GABAergic system functions is significantly correlated with social and communicative impairment and the disproportionate effect between excitatory and inhibitory neurotransmission in the context of ASD, which causes core symptoms of ASD [53].

Glutamate and GABA, the brain's primary excitatory and inhibitory neurotransmitters, are tightly interlinked through the astrocyte-mediated glutamate–glutamine–GABA cycle. After glutamate is released from neurons during synaptic transmission, it is rapidly taken up by surrounding astrocytes. Within these astrocytes, glutamate is converted to glutamine via glutamine synthetase, an enzyme exclusively expressed in astrocytes, and then shuttled back to neurons. Neurons subsequently convert this glutamine back into glutamate and, in GABAergic neurons, further into GABA via glutamate decarboxylase [54],[55]. This metabolic interdependence results in a disorder in one aspect of the cycle, such as a deficiency of glutamate or glutamine, which can disrupt not only the balance of glutamate but also that of GABA so that the balance between excitatory or inhibitory neurotransmission, which is vital for normal brain function, can be affected [56],[57]. Astrocytes are essential not only for transmitter recycling but also for neurotransmission homeostasis, energy metabolism, and nitrogen balance, emphasizing that proper cognitive and neural network function relies on the precise coupling of the glutamatergic and GABAergic systems [58],[59].

## 4. Computational approaches to E/I balance

Advances in computational methods and machine learning have significantly enhanced the study of the complex E/I imbalance hypothesis in ASD. These approaches quantify E/I ratios using biomarkers such as the Hurst exponent (H), derived from resting-state fMRI or EEG data, which correlates with the long-range temporal correlation of neural activity. Smaller H values indicate a predominance of excitation over inhibition, a characteristic profile observed in individuals with ASD [60]–[62]. For example, neuronal network models demonstrate that manipulating structural E/I ratios directly influences functional E/I measures, producing an inverse U-shaped relationship between E/I balance and long-range temporal correlations (LRTCs) [62]. Algorithms utilize oscillation amplitudes and LRTC covariance to calculate the functional E/I ratio (fE/I), validated in healthy populations where GABAergic enhancers reduce fE/I and in ASD cohorts, which exhibit increased fE/I variability and altered LRTC patterns [63]. Machine learning classifiers, such as random forests, leverage these metrics for diagnostic purposes, distinguishing ASD from schizophrenia with AUCs up to 84% by integrating H values from 53 brain regions alongside phenotypic data (e.g., ADOS and PANSS scores) [60].

Beyond diagnosis, computational frameworks elucidate ASD heterogeneity by linking E/I dysregulation to genetic, molecular, and network-level factors. Models incorporating neurotransmitter dynamics (e.g., GABA and glutamate) reveal subgroup-specific E/I patterns that reconcile contradictory findings of increased excitation or inhibition in ASD [63]. Notably, sex differences in the expression of E/I-related genes, particularly in social brain regions such as the ventromedial prefrontal cortex, contribute to variation in symptom severity and treatment response [61],[64]. These insights guide therapeutic strategies, including bumetanide and transcranial direct current stimulation (tDCS), which computationally predict normalization of E/I ratios. Post-intervention EEG reveals increased signal complexity, as measured by entropy, and reduced pathological low-frequency connectivity correlating with behavioral improvements [64]. Nonetheless, challenges persist, including inconsistency in MRS-based neurotransmitter measurements and the need to generalize models across diverse datasets. Future work should focus on integrating multimodal genetic, imaging, and clinical data to enable personalized E/I-targeted interventions [64].

### 4.1. Modeling frameworks

Neuroscientific modeling frameworks span multiple scales of abstraction, balancing biological fidelity with computational efficiency. Biophysically detailed neuron models, exemplified by Hodgkin–Huxley (HH) formulations and conductance-based approaches, explicitly simulate ion channel dynamics and membrane potentials. These models capture nonlinear interactions such as action potential generation and synaptic integration through differential equations representing ionic currents (e.g., *I_Na_ = g_Na_m^3 h (V-E_Na_*) for sodium currents) [65],[66],

where:

- *I_Na_* is the sodium current,
- *g_Na_* is the maximal sodium conductance,
- *m* is the activation gating variable,
- *h* is the inactivation gating variable,
- *V* is the membrane potential, and
- where *E_Na_* is the sodium reversal potential.

Spiking neural networks (SNNs) simplify the process of neuronal communication to discrete instances of spike timing and can be implemented efficiently in neuromorphic hardware [67], although their biological verisimilitude ranges between simplified integrate-and-fire neurons and multicompartmental HH models [68]. In large-scale network simulations, mean-field and neural mass models can be further simplified by modeling populations of neurons as coupled nonlinear differential equations governing the ensemble rate of firing, the dynamics of synapses and the emergence of synchronization phenomena [69],[70]. For example, a mean-field approach developed by Carlu et al. [69] can correctly model asynchronous irregular regimes within conductance-based neuronal networks, whereas Rodrigues et al. [70] performed systematic connections between macroscopic neuronal masses and scaled conductance-based neuron processes. These strategies are increasingly incorporated into computational frameworks: BrainCog is a system used to implement SNN-based brain simulation and AI [71], and osNEF is a computational framework that builds a functional cognitive system using biophysically detailed neurons through oracle-supervised learning [65]. A cross-paradigm integration example would be the reconfigurable neuromorphic systems of both SNNs and mean-field-like convolution/reservoir computing that would answer trade-offs between biological realism and scalability [72]. Limitations include parameter sensitivity in detailed models and abstraction gaps in reduced models, driving ongoing innovations such as multitask learning for predicting HH model behaviors and adaptive mean-field formulations for electrical stimulation responses [73].

### 4.2. Simulation platforms and tools

Biophysically realistic simulation frameworks such as NEURON, Brian, NEST, and the virtual brain (TVB) are highly applicable in computational studies of E/I balance in autism because the algebraic disruption of network-level mechanisms is investigated. NEURON is good at modeling in detail the dynamics of individual neurons and synapses so that it can be used to investigate how ion channelopathies or modifications of synaptic receptors (e.g., NMDA/GABA imbalances) disrupt E/I ratios in mutations associated with autism [74],[75]. Brian, a spiking neural network framework based on Python, supports large-scale simulations of E/I-balanced networks, such as those studies that normalized atypical firing patterns in autism models by tuning inhibitory synapses [76]. The parallelized simulation of cortical microcircuits could be performed with the use of NEST, which models the effects of E/I imbalance on population synchrony and information transfer, especially in those networks that are affected by the parvalbumin interneuron loss that is typical of autism [77]. TVB integrates whole-brain connectomics with local neural mass models, revealing how region-specific E/I disruptions alter global functional connectivity in autism, such as reduced long-range inhibition in prefrontal–auditory pathways (Table 2) [78]–[80]. Future work requires hybrid models coupling TVB's macroscale dynamics with NEST/Brian's microscale precision to map E/I trajectories across neurodevelopment in autism.

**Table 2.** Overview of computational modeling tools for studying E/I imbalance in autism ASD.

**Tool**	**Key Features**	**Autism-Specific Applications**	**Limitations**	**Ref**
NEURON	Biophysical modeling with Hodgkin-Huxley formalism for simulating ion channels and synapses.	Simulates cellular-level E/I imbalances such as reduced GABAergic inhibition in valproate-induced autism models. Models' genetic effects like CYFIP1 mutations affecting synaptic scaffolding.	Computationally expensive, not scalable to whole-brain networks.	[81],[82]
Brian/Brian2	Spiking neural networks with Python interface. Supports AdEx neurons and GPU acceleration for efficient simulation.	Reveals E/I imbalances increase neural noise, modeling sensory hypersensitivity. Useful for rapid testing of circuit-level hypotheses.	Limited support for mean-field models; custom code often required for scaling.	[83]
NEST	Massively parallel simulations of heterogeneous, large-scale spiking networks. Integrates with mean-field modeling.	Models' autism-linked gene mutations (e.g., Fmr1, Cntnap2) affecting cortical flexibility and gamma oscillations. Enables network-level analysis of disrupted E/I dynamics.	Less detailed at the single-cell biophysical level.	[84]
The Virtual Brain (TVB)	Multiscale brain simulations using neural mass/mean-field models. Personalized with individual MRI data.	Simulates macroscopic E/I ratios across brain regions. Links increased global E/I to fMRI abnormalities in ASD. Supports stratification of ASD subtypes through model personalization.	Lacks cellular resolution; requires integration with tools like NEURON or NEST for full-scale validation.	[85]

## 5. Neurotransmitter dynamics in ASD models

### 5.1. Serotonergic system in ASD

**Figure 6.**
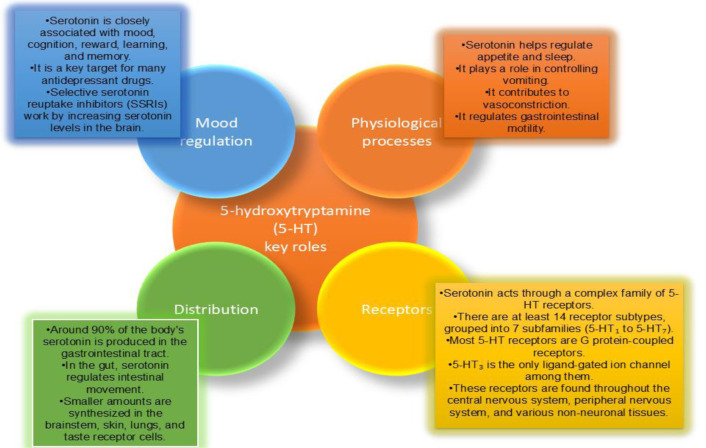
Key roles of 5-hydroxytryptamine (5-HT) in mood regulation, physiological processes, receptor diversity, and body distribution.

The serotonergic system is a complicated system of neurons that produces, releases, and detects serotonin (5-hydroxytryptamine or 5-HT), a major neurotransmitter that plays an important role in the regulation of several physiological and behavioral processes. Figure 6 visually summarizes this complex system, illustrating the synthetic pathway, release mechanisms, and crucial reuptake process. Approximately 90% of the body's serotonin is produced in the gastrointestinal tract, with fewer quantities found in the brainstem, especially in the raphe nuclei, as well as in the skin, lungs, and taste receptor cells [86]. In the central nervous system, serotonin affects mood, thoughts, hunger, sleeping, vomiting, vasoconstriction, and touch-feel. It exerts its effects through a diverse array of receptors, with at least 14 subtypes classified into seven families, known as 5-HT_1_ to 5-HT_7_ (Table 3) [87]. Most of these receptors are G protein-coupled, except for 5-HT₃, which functions as a ligand-gated ion channel. These receptors are widely distributed across the brain, peripheral nervous system, and various nonneuronal tissues. The serotonergic system is a major target for pharmacological intervention, especially in the treatment of psychiatric disorders, with drugs such as selective serotonin reuptake inhibitors (SSRIs) designed to increase serotonin levels and activity [88].

**Table 3.** Serotonin receptor subtypes and their roles in ASD-related neurodevelopment.

**Receptor subtype**	**Primary function**	**ASD relevance**
5-HT1A	Neurite outgrowth, dendritic pruning	Altered in raphe nuclei; impacts cortical connectivity 6
5-HT2A	Synaptic plasticity, spine morphology	Linked to sensory processing abnormalities; therapeutic target 9
5-HT3	GI motility, neurotransmitter release	Mediates gut-brain signaling; microbiota interaction point 15
5-HT7	Neuronal migration, synaptic maturation	Critical during fetal development; disrupted in ASD models 12

### 5.2. Dopaminergic system in ASD

The dopaminergic system shows dramatic changes in ASD, which are characterized not only by genetic disorders but also by neurotransmitter imbalances and circuit-level abnormalities. Genetic factors are important in the modulation of dopaminergic signaling, and mutations related to ASD have been associated with the disruption of genes encoding DA receptors (e.g., *DRD1–DRD5*), synthetic enzymes (THs), and transporters (DAT/SLC6A3) [89],[90]. The copy number variations (CNVs) in these loci are associated with social deficits and repetitive behaviors, suggesting that dopaminergic dysregulation is a core pathological mechanism [91]. Neurotransmitter dynamics are associated with codependent dysregulation: disturbed E/I balance elevates striatal glutamate, which hyperactivates mesocortical DA transaction, whereas GABAergic deficiency fails to provide tonic inhibition of dopaminergic neurons [53],[78],[92]. This imbalance is evidenced by abnormal DA metabolite levels in ASD cohorts and altered DA-dependent plasticity in *Shank3* mutant models [93].

These genetic and neurotransmitter interactions are incorporated into computational models to model dopaminergic dysfunction. The striatal–cortical loop has the disadvantage of showing that lower dopaminergic tone disrupts reinforcement and cognitive flexibility in prefrontal areas, but hyperactivity of the ventral tegmental area (VTA) is causative of repetitive behaviors [89],[91]. These models integrate apparently paradoxical observations, such as prefrontal hypodopaminergia against hyperdopaminergia in striatal circuits, via a feedback loop involving glutamatergic inputs and inhibitory GABAergic interneurons [94]. Imaging data corroborate these findings, showing disrupted functional connectivity between the VTA and frontostriatal networks in ASD patients.

### 5.3. Glutamatergic and GABAergic systems in ASD

The E/I imbalance hypothesis: This hypothesis assumes that a change in glutamatergic-GABAergic signaling provides the foundation of the key behavioral phenotype in ASD. There is considerable evidence in the direction of abnormal neurotransmitter dynamics, with separate studies continuing to show increased levels of glutamate and decreased concentrations of GABA in most areas of the cortex both in postmortem studies and in findings of biomarkers [53],[95],[96]. For example, Alabdali et al. reported substantial changes in the glutamate/GABA ratios and metabolism of glutamine and GABAergic enzymes in both ASD patients compared with controls, indicating improper recycling of neurotransmitters at the peak of their stimulating intensity [53]. These results correlate with the results of competitive gene set analysis by Hollestein et al., who reported that glutamatergic/GABAergic gene expression predicts the magnitude of symptoms and cortical thickness impairments [78].

Mechanistically, in animal models, genetic perturbations exacerbate E/I imbalance through multiple pathways:

- Valproate models in rats exhibit the downregulation of *Gabra1* and *Gabrb3* as well as the upregulation of NMDA receptor subunits in the hippocampus [97].
- *Shank3* knockout mice exhibit reduced GABA receptor densities in thalamocortical circuits [98].
- Developmental deficits in GABAergic interneurons disrupt sensory processing, as shown by impaired dorsal root ganglion responses in Gabrb3-deficient mice [99].

**Figure 7.**
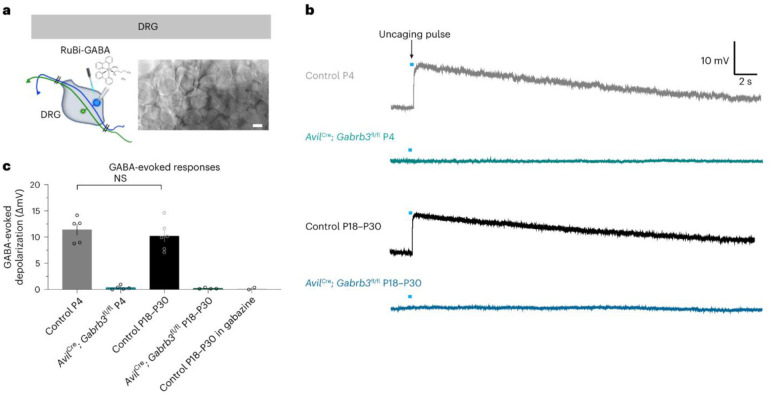
Impaired GABAergic signaling in Gabrb3-deficient mouse models. (a) Schematic of dorsal root ganglion (DRG) stimulation with RuBi-GABA. (b) Representative traces of GABA-evoked responses showing markedly reduced responses in Gabrb3-deficient mice at both the early (P4) and late (P18–P30) postnatal stages. (c) Quantification of GABA-evoked depolarization, highlighting impaired GABAergic signaling in mutant animals. These results support the hypothesis that GABAergic dysfunction contributes to E/I imbalance in ASD (adapted from [99] under the CC-BY 4.0 license).

Figure 7. E/I Imbalance Hypothesis in ASD. This figure graphically encapsulates the central hypothesis that autism spectrum disorder is characterized by a shift in excitation/inhibition balance, specifically marked by reduced GABAergic (inhibitory) signaling and elevated glutamatergic (excitatory) activity. The depicted E/I imbalance is mechanistically traced to essential molecular and genetic defects, including: 1) downregulation of GABA receptor subunits such as Gabra1 and Gabrb3, resulting in decreased inhibitory tone; 2) insufficient levels of the synaptic scaffolding protein SHANK3, leading to disrupted synaptic integrity; and 3) alterations in NMDA receptor function affecting excitatory neurotransmission. Collectively, the figure demonstrates how these convergent molecular disturbances drive network-level E/I imbalance, which in turn underpins the severity of core ASD symptoms and widespread disorganization of brain circuit architecture.

There is a contradiction concerning directionality, whereby in studies conducted using MRS, some have reported that GABA levels are higher in autistic children [100]; however, some researchers have reported that GABA levels are deficient. This heterogeneity may be due to methodological discrepancies, as the development or subtypes of ASD with different and unique GABA/glutamate changes over time [78]. Genetic modifiers further complicate these landscape polymorphisms in *GRIN2A*, *GABRB3*, and glutamate transporter genes (*SLC1A1*), which are correlated with symptom severity but exhibit inconsistent expression patterns across brain regions [101].

### 5.4. Compensatory and homeostatic mechanisms in E/I balance during development

During neural development, the maintenance of E/I balance relies on compensatory and homeostatic mechanisms that stabilize activity despite dynamic changes in connectivity. Homeostatic plasticity ensures that neurons adjust their excitability and synaptic strength to maintain stable firing rates, even as circuits undergo extensive remodeling [102]. For example, developing spinal networks show compensatory changes in AMPA receptor expression and GABAergic signaling when activity is perturbed, indicating that GABA participates in activity sensing during early development [103]. Similarly, cortical circuits regulate interneuron population size via activity-dependent apoptosis, providing an early homeostatic checkpoint that aligns inhibitory capacity with overall network excitability [104]. Rapid compensatory plasticity has also been observed in vivo, where inhibitory plasticity dynamically reduces correlated excitatory activity during strong stimulation, highlighting fast-acting stabilizing mechanisms [105]. At the molecular level, mechanisms such as retinoic acid-mediated synaptic scaling fine-tune both excitatory and inhibitory transmission, linking developmental homeostasis to later vulnerability in disorders such as autism and Fragile X syndrome [106]. Moreover, regional specificity, such as in the ventral hippocampus, shows that brain areas with high excitability may rely on strong compensatory mechanisms to resist imbalances, offering a model for understanding neurodevelopmental disorders [107]. Taken together, evidence suggests that compensatory and homeostatic mechanisms act across molecular, cellular, and network levels to maintain the E/I balance during development and that their failure or dysregulation can contribute to long-term vulnerability to neuropsychiatric disease.

### 5.5. Contributions to ASD identification and management

E/I balance computational modeling has made a profound contribution to the identification and management of ASD because of its ability to connect neurotransmitter dynamics and genetics to measurable biomarkers. Neural dynamics modeling indicates that ASD can be stratified using aberrant E/I ratios, especially glutamatergic hyperactivity and GABAergic deficits, and are strong electrophysiological ASD biomarkers. For example, Bruining et al. demonstrated that critical brain dynamics can quantify a functional E/I ratio (fE/I) from neuronal oscillations, with ASD cohorts exhibiting abnormal power-law distributions in resting-state EEGs that correlate with symptom severity [62]. This strategy offers a noninvasive biomarker for early screening, validated by Tang et al.'s work in BTBR mice, which revealed a decrease in parvalbumin-positive interneurons and glutamate/GABA imbalances in the circuits of the auditory cortex [108]. Genetically informed models also enhance identification; Hollestein et al. incorporated transcriptomic information to demonstrate that a gene set of glutamate (GRM, GRIN families) and GABA (GABR) genes differentially correlates with cortical thickness anomalies, as well as social communication deficits; therefore, genetic subgroups of patients with ASD are possible via this method of identification [78].

For management, these models highlight precision pharmacological targets. Dysregulated neurotransmitter receptors such as downregulated GABA_A subunits and hyperactive NMDA receptors identified in genetic mouse models [109] inform trials of GABA enhancers (e.g., arbaclofen) and glutamate modulators (e.g., memantine) [110]. Model frameworks such as neurotransmitter kinetics to functional connectivity with computational models such as the multiscale dynamic mean field (MDMF) model developed by Naskar et al. allow prediction of how E/I-correcting agents revert abnormalities at the network scale (e.g., hypoconnectivity of the default mode network) [111]. Neuroimaging-based management is also emerging: MRS studies consistently report elevated prefrontal GABA in adults with ASD [112], whereas pediatric cohorts show age-dependent GABA reductions in parietal regions [113], suggesting the need for age-stratified treatment protocols.

Nevertheless, serious gaps exist. There are conflicting data, i.e., some models show GABAergic excess as opposed to deficiency data, which illustrates the heterogeneity of ASD. Genetic mutations (e.g., SLC6A1 and SHANK3) have pleiotropic effects on the E/I balance, complicating target selection [112]. Future research must prioritize longitudinal models incorporating DA–serotonin crosstalk, develop noninvasive fE/I monitoring tools for real-time treatment adjustment and validate cross-species biomarkers through collaborative consortia such as the AGRE. Integrating multiomics data into neural mass models will enable dynamic, personalized E/I modulation strategies.

## 6. Genetic factors influencing E/I balance

### 6.1. Genetic mutations affecting synaptic proteins

Genetic mutations in synaptic scaffolding proteins, particularly SHANK3, profoundly disrupt the E/I balance in neural circuits, a core pathophysiological mechanism in ASD. SHANK3 mutants play a dysfunctional role and abnormally form glutamatergic synapses by destabilizing the postsynaptic density (PSD) to produce inhibited AMPA receptor-mediated currents and minimal long-term potentiation (LTP) in the hippocampal and prefrontal networks [114],[115]. This results from disrupted SHANK3-dependent scaffolding of glutamate receptors (e.g., AMPA, NMDA) and downstream signaling effectors such as GKAP and Homer [116],[117]. Importantly, SHANK3 mutations have also been shown to disrupt inhibitory synapses: In mice, deletion of exon 9 results in decreased GABAergic transmission into the hippocampus and striatum because of inappropriate gephyrin clustering and GABA receptor trafficking [118],[119]. This dual impairment exacerbates excitation via weakened inhibitory neurotransmission, shifting the E/I balance toward hyperexcitability, manifesting as ASD-like behaviors, including social deficits and repetitive actions [120].

Furthermore, mutation of SHANK3 weakens structural synapses. Compared with ultrastructural analyses of the prefrontal cortex of Shank3-deficient models, asymmetric synapses and the immaturity of dendritic spines are intercorrelated with a low thickness of the PSD and defects in vesicle docking [121]. These defects intersect with those of the neuroligin (NLGN) and neurexin (NRXN) pathways: Knockdown of NLGN-1 decreases the density of excitatory synapses, whereas NLGN-2 mutations have a poor bias toward inhibitory synapse function [122]. This synaptic imbalance extends to neuroinflammation and redox dysregulation, as SHANK3 mutations increase nitrosative stress markers (e.g., S-nitrosylated proteins) and proinflammatory cytokines, such as IL-6, further exacerbating circuit hyperexcitability [123],[124].

The resulting E/I mismatch changes network synchronization, reflected in the disturbance of gamma oscillations in the network of corticostriatal connections in Shank3-deficient mice [125]. Interestingly, secondary responses can be developed: Compensatory models in which SHANK3-deficient neurons overexpress SHANK2 recover from synaptic dysfunction, indicating functional redundancy between SHANK family members [126]. However, mutations affecting multiple synaptic genes (e.g., concurrent disruptions in NRXN1, NLGN3, and SHANK3) amplify E/I dysregulation, reflecting a “synaptopathy axis” in ASD pathogenesis [127],[128].

### 6.2. Alterations in glutamatergic and GABAergic signaling

The imbalance of E/I is one of the fundamental pathophysiological processes in ASD, and genetic variations in how glutamate and GABA neurotransmitters (belonging to different classes) bind to their respective receptor subunits are defined as contributing to E/I imbalance. Reduced expression of GABAergic genes, particularly GABRB3 and GABRA5, which encode GABAA receptor subunits, compresses inhibitory neurotransmission. In valproic acid (VPA)-induced ASD rat models, significant downregulation of *GABRB3* expression in the cerebral cortex and cerebellum is correlated with behavioral deficits [97],[129]. GABAergic dysfunction reduces inhibitory postsynaptic currents (IPSCs), which impair cortical circuit synchronization and neuronal hyperexcitability [130].

On the other hand, the dysregulation of glutamatergic signaling is the result of SNPs in GRIN2B (NMDA receptor subunit GluN2B) and GRIA2 (AMPA receptor subunit GluA2). Human studies have revealed altered GRIN2B splicing and trafficking deficits in ASD, increasing NMDA receptor-mediated calcium influx and excitotoxic vulnerability [131]. Cortical expression studies have revealed that cortical regional disturbances, e.g., overexpression of GRIA2 in sensory regions, lead to sensory cortical hypersensitivity (e.g., sensory hypersensitivity with increased glutamate + glutamine levels) [132]. These alterations shift the E/I ratios toward excitation, as quantified by:

- Reduced GABAA receptor clustering in hippocampal neurons
- Enhanced glutamatergic synaptic transmission
- Impaired long-term potentiation (LTP) thresholds

This imbalance of E/I affects most of the neurodevelopmental mechanisms involved, such as synaptic pruning and network remodeling, which form the basis of the behaviors associated with ASD, such as sensory hypersensitivities and impaired social affairs [133].

Integrative models propose bidirectional dysregulation: glutamate-driven hyperexcitation combined with GABAergic hypofunction creates pathological feedback loops. Neuroinflammation exacerbates this imbalance, as proinflammatory cytokines directly suppress GABA synthesis enzymes (e.g., GAD67) and increase glutamate release from microglia [95].

### 6.3. Glutamatergic dysregulation

#### 6.3.1. NMDA/AMPA receptor variants

E/I imbalance caused by genetic mutations in glutamatergic receptor subunit DNA, such as *GRIN2A* (which encodes the glutamate NMDA receptor subunit GluN2A) and *GRIA* (which encode AMPA receptor subunits), affects the E/I balance by exaggerating excitatory postsynaptic currents and different physiologies in synapses. Loss-of-function mutations in GRIN2A inhibit the activity of NMDA receptors, causing compensated hyperexcitation of AMPA-driven circuits and disrupted network oscillations of the hippocampus that are essential in working memory, as shown in Grin2a mutant mice with deficits in spatial memory and disturbed gamma oscillations [134]–[136]. Moreover, pathogenic mutations in GRIA (e.g., GRIA2) lead to increased calcium permeability of AMPA receptors, resulting in long-term excitatory neuron stimulation and synapse destabilization, contributing to E/I imbalance [137]. These dysfunctions are clinically associated with cognitive impairment in schizophrenia patients, with postmortem studies demonstrating low expression of GRIN2A in the dorsolateral prefrontal cortex and AMPA receptor trafficking in both schizophrenic patients and controls [133]. Genetic associations further underscore this link, with GRIN2A variants conferring high schizophrenia risk (odds ratio >20 for specific mutations) [138].

#### 6.3.2. Synaptic scaffolding proteins

Synaptic scaffolding proteins, including the SHANK3, PSD-95, ProSAP, and NLGN/NRXN families, are critical architectural and functional elements at excitatory synapses, governing glutamate receptor trafficking, synaptic stability, and plasticity. Mutations in genes encoding these proteins (e.g., SHANK3, NLGN3/4, and NRXN1) are strongly implicated in ASD, frequently disrupting the E/I balance [94],[127],[139]. Computational models incorporating these disruptions revealed that *SHANK3* haploinsufficiency reduces AMPA and NMDA receptor mobility at postsynaptic densities, impairing long-term potentiation (LTP) and glutamatergic signaling fidelity [126]. This aligns with in vivo observations of dendritic spine dysgenesis and weakened cortical–striatal synaptic transmission in *Shank3*-deficient rodents, which exhibit ASD-like social deficits and repetitive behaviors [140]. Crucially, scaffolding proteins such as SHANK3 physically integrate NMDA receptors with metabotropic glutamate receptors (mGlu5) via PSD-95-Homer complexes, forming a “glutamate receptosome” whose destabilization in ASD models abolishes activity-dependent synaptic strengthening [141].

Contradictory evidence appears in the issue of the specificity of E/I disruptions. Although certain studies have revealed mostly glutamatergic deficits [92], cooccurring GABAergic dysregulation, such as diminished gephyrin clustering at inhibitory synapses in Shank3 models, has been reported [139]. This duality underscores the need for multiscale modeling, where the dynamics of pre- and postsynaptic scaffoldings are simulated concurrently at the stages of excitatory and inhibitory networks. Synaptic connections are sometimes not considered in the astrocytic modulation of scaffolding proteins [142], although it has been demonstrated that astrocyte-secreted proteins control SHANK3 localization and turnover in synapses [143]. Additionally, most frameworks overlook isoform-specific effects; for example, SHANK3 exon-specific deletions yield distinct electrophysiological and behavioral phenotypes in mice [144], though computational studies rarely incorporate such molecular diversity.

## 7. Integrated perspective: Neurotransmitters and genetic interactions

ASD is a complicated neurodevelopmental disorder that has both hereditary and environmental risk factors [145]. New studies have highlighted the importance of genetic alternatives, such as copy number variants (CNVs) and single nucleotide polymorphisms (SNPs), in disrupting neurodevelopmental pathways [146]. These genetic differences influence multiple neurotransmitter systems, mostly the GABAergic and glutamatergic systems, which is one of the causes of excitation/inhibition imbalance in people with ASD [112]. Research indicates that dysfunctions in the production, secretion, and distribution of neurotransmitters, including oxytocin, also contribute significantly to the development of ASD [147]. While numerous genes have been linked to ASD, they account for only a fraction of cases, underscoring the etiological heterogeneity of the disorder [148],[149].

The interplay between genetic susceptibility and neurotransmitter dysregulation is a critical area of investigation [150]. For example, studies have shown that mutations in the genome or in the GABA receptor or serotonin transporter might affect the functions of these neurotransmitter systems by being a cause of the ASD phenotypes [151]. A few studies have reported increased levels of prefrontal GABA in adults with ASD [112], and fewer studies have reported decreased glutamate concentrations in certain areas of the brain [112]. These inconsistencies highlight the complexity of neurotransmitter involvement in ASD and the need for further research to clarify the roles of different neurotransmitters in various brain regions and developmental stages [152].

Although it is now possible to identify genetic and neurotransmitter abnormalities, there are large gaps in the knowledge of how these factors interact to produce ASD [153]–[155]. Future studies must be aimed at combining genetic, neuroimaging, and behavioral data to understand the etiology of ASD more extensively [156]. Researchers conducting longitudinal studies should focus on exploring the effects of genetic expression on neurotransmitter function during developmental stages, and further investigations of patient characteristics are needed [157]. In addition, the use of epigenetic modifications and environmental factors in the regulation of the expression of genes and neurotransmitters can shed some light on the pathogenesis of ASD [146]. Advanced techniques such as single-cell genomics and transcriptomics, combined with ASD-specific organoid models, hold promise for revealing novel mechanistic pathways and therapeutic targets [158]. It is also important to consider sex differences in ASD, as males are more frequently diagnosed, suggesting potential phenotypic and camouflaging differences between the sexes [159]. Addressing these gaps will facilitate the development of more effective diagnostic and therapeutic strategies for ASD [160]. An integrated overview of neurotransmitter systems, associated genes, functions, and related disorders is presented in Table 4, which highlights the complex genetic and neurochemical interactions involved in ASD and related conditions.

**Table 4.** Integrated overview of neurotransmitter systems and associated genetic interactions.

**Neurotransmitter System**	**Key Genes Involved**	**Function of Gene Products**	**Associated Disorders**	**Mechanistic Insight**	**Ref**
Dopaminergic	DRD1–5, SLC6A3 (DAT1), TH, DDC, COMT, MAO	Dopamine (DA) receptors (DRD1–5) mediate DA signaling; DAT1 regulates DA reuptake; TH and DDC are involved in DA synthesis; COMT and MAO are involved in DA degradation.	Parkinson's disease, ADHD, addiction, restless legs syndrome	Degeneration of dopaminergic neurons in the substantia nigra leads to motor deficits in Parkinson's disease. Aberrant DA signaling in the mesolimbic pathway contributes to positive symptoms of schizophrenia. Variations in DAT1 influence susceptibility to ADHD.	[161]–[164]
Serotonergic	HTR1A–7, SLC6A4 (SERT), TPH1/2, MAO	Serotonin receptors (HTR1A–7) mediate serotonin signaling; SERT regulates serotonin reuptake; TPH1/2 are involved in serotonin synthesis; MAO is involved in serotonin degradation.	Depression, anxiety disorders, obsessive-compulsive disorder (OCD), ASD	Dysregulation of serotonin signaling contributes to mood disturbances in depression and anxiety. SERT polymorphisms influence vulnerability to stress-related disorders. Serotonin dysregulation can affect brain E/I balance.	[163]–[167]
Glutamatergic	GRIA1–4 (AMPA receptors), GRIN1, GRIN2A-D, GRIN3A (NMDA receptors), GLUL, SLC1A2 (EAAT2)	AMPA and NMDA receptors mediate excitatory neurotransmission; GLUL is involved in glutamate synthesis; EAAT2 regulates glutamate reuptake.	Alzheimer's disease, epilepsy, stroke, traumatic brain injury.	Excitotoxicity, resulting from excessive glutamate signaling, contributes to neuronal damage in stroke and traumatic brain injury. NMDA receptor dysfunction is implicated in the pathophysiology of schizophrenia. Glutamatergic dysfunction can affect the brain's E/I balance.	[168]–[172]
GABAergic	GABRA1–6, GABRB1–3, GABRG1–3 (GABAA receptors), GABBR1–2 (GABAB receptors), GAD1/2, SLC6A1 (GAT1)	GABAA and GABAB receptors mediate inhibitory neurotransmission; GAD1/2 are involved in GABA synthesis; GAT1 regulates GABA reuptake.	Anxiety disorders, epilepsy, insomnia, schizophrenia	Reduced GABAergic inhibition contributes to seizures in epilepsy. GABAA receptor dysfunction is implicated in anxiety disorders. MicroRNAs affect GABAergic synapse function in Alzheimer's disease.	[173]–[175]
Cholinergic	CHRNA1–10 (nicotinic acetylcholine receptors), CHRM1–5 (muscarinic acetylcholine receptors), CHAT, ACHE	Nicotinic and muscarinic receptors mediate cholinergic signaling; CHAT is involved in acetylcholine synthesis; ACHE breaks down acetylcholine.	Alzheimer's disease, myasthenia gravis, schizophrenia	Loss of cholinergic neurons in the basal forebrain contributes to cognitive decline in Alzheimer's. Autoimmune destruction of acetylcholine receptors leads to muscle weakness in myasthenia gravis. Cholinergic dysfunction can affect the brain's E/I balance.	[174],[176]
Histaminergic	HRH1-4, HDC	Histamine receptors (HRH1–4) mediate histamine signaling; HDC is involved in histamine synthesis.	Narcolepsy, schizophrenia, Tourette Syndrome	Deregulation of histamine-related gene expression may play a role in.	[177]

### 7.1. Gut–brain axis integration in E/I balance models

The gut microbiome significantly influences neurotransmitter function, a connection that has profound implications for ASD symptomatology via the intricate gut–brain axis [178]. This bidirectional communication system involves immune, endocrine, and neural pathways, with the gut microbiota playing a pivotal role in modulating central nervous system activity and behavior. Dysbiosis, an imbalance in the gut microbial community, is frequently observed in individuals with ASD and is associated with alterations in key neurotransmitters, such as GABA, serotonin (5-HT), and DA, which are crucial for regulating mood, cognition, and social behaviors [179].

Specifically, the gut microbiota can synthesize and metabolize neuroactive compounds and their precursors. For example, tryptophan, a precursor to serotonin, is influenced by microbial activity, impacting its systemic availability and subsequent activity in the brain. Serotonin dysregulation is commonly implicated in ASD, with gut-derived serotonin potentially affecting brain development and function. Similarly, an imbalance in GABAergic and glutamatergic markers has been noted in ASD, contributing to abnormal neural excitability. The gut microbiota can influence the production of short-chain fatty acids (SCFAs), such as butyrate, propionate, and acetate, which can cross the blood–brain barrier and modulate neuroinflammation and neurotransmitter synthesis. For example, studies suggest altered levels of propionate, a metabolite that can impact brain function and behavior, in individuals with ASD [180].

Moreover, microbial dysbiosis can compromise the integrity of the intestinal epithelial barrier, leading to increased gut permeability, often referred to as “leaky gut”. This increased permeability allows microbial metabolites, toxins, and inflammatory signals to enter the bloodstream and subsequently the brain. These systemic factors can trigger neuroinflammation and alter neurotransmitter signaling pathways critical for neurodevelopment, thereby contributing to the complex behavioral and cognitive deficits observed in ASD. Studies have shown that alterations in the gut microbiota are linked to immune dysregulation and inflammation, which are significant comorbidities in individuals with ASD [181].

The interrelationship among the gut microbiota, inflammation, and neurological symptoms is evident. As illustrated in the diagram, microbiota composition, inflammatory responses, and signal transduction pathways are common factors affecting both ASD and cancer, emphasizing the systemic impact on gut health [182]. This highlights that disruptions in these pathways can significantly contribute to the development of ASD.

Despite growing evidence, inconsistencies exist across studies regarding the specific microbial taxa involved and the precise mechanisms by which they influence neurotransmission in ASD. This variability can be attributed to the diverse study designs, geographical locations, age groups, and genetic backgrounds of the participants, as well as the heterogeneous nature of ASD. For example, recent metagenomic sequencing studies have identified alterations not only in bacterial components but also in archaea, fungi, and viruses in the gut of children with ASD, indicating more complex multikingdom dysbiosis than previously understood [183]. The interplay between these microbial kingdoms and their impact on host physiology, including neurotransmitter pathways, requires further elucidation.

**Figure 8.**
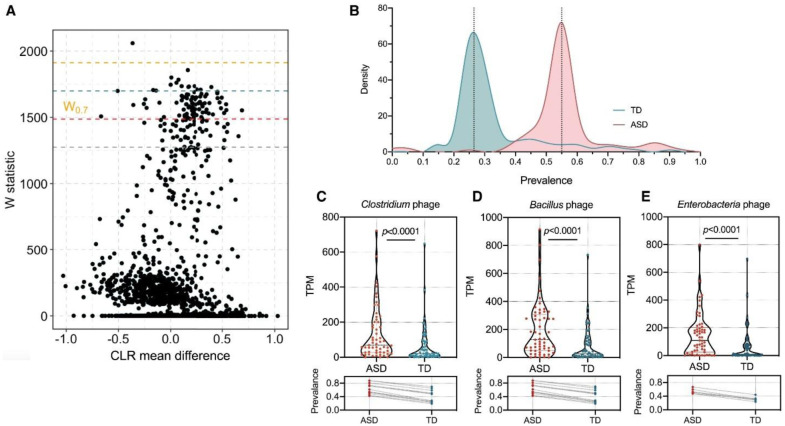
Enrichment of Clostridium, Bacillus, and Enterobacteria phages in children with ASD. (A) Analysis of the composition of microbiome (ANCOM) results comparing viral-like sequence (VLS) abundances between children with ASD and typically developing (TD) children, adjusted for covariates including age, sex, and BMI. A threshold of W > 0.7 indicates significant differential abundance. (B) Distribution density plot of 177 differentially abundant VLSs, showing a greater prevalence in the ASD (red) group than in the TD (blue) group. (C–E) Dot plots illustrating the differences in the abundance and prevalence of viral sequences annotated to Clostridium phage (C), Bacillus phage (D), and Enterobacteria phage (E) between the ASD and TD groups, revealing statistically significant enrichment of these phages in ASD children (p < 0.0001) (adapted from [184] under the CC-BY 4.0 license).

Wan et al. (2024) reported significant enrichment of Clostridium, Bacillus, and Enterobacteria phages in the gut virome of children with ASD compared with typically developing children on the basis of analysis of composition of microbiome (ANCOM) results adjusted for age, sex, and BMI (Figure 8). They identified 177 differentially abundant viral-like sequences (VLSs) that were predominantly enriched in ASD, with dot plots showing a marked increase in the abundance and prevalence of these specific phages in ASD children (p < 0.0001). This enrichment reflects disrupted viral ecology and altered bacteriophage–bacterial interactions that may impair microbial pathways linked to neuroactive metabolite biosynthesis, thus potentially contributing to ASD pathogenesis [184].

The involvement of the gut microbiome in ASD is not limited to symptom manifestation but also extends to potential diagnostic and therapeutic avenues. Altered gut microbiota profiles are increasingly being considered potential biomarkers for ASD, particularly in early childhood, when interventions might be most effective [185]. Therapeutic strategies focused on modulating the gut microbiota, such as fecal microbiota transplantation (FMT), probiotics, prebiotics, and microbiota-directed foods (MDFs), show promise in ameliorating ASD-associated symptoms by restoring microbial balance and optimizing neurotransmitter function. In particular, FMT improves cognitive and gastrointestinal symptoms in ASD patients, suggesting a direct link between microbial composition and clinical outcomes [186].

**Figure 9.**
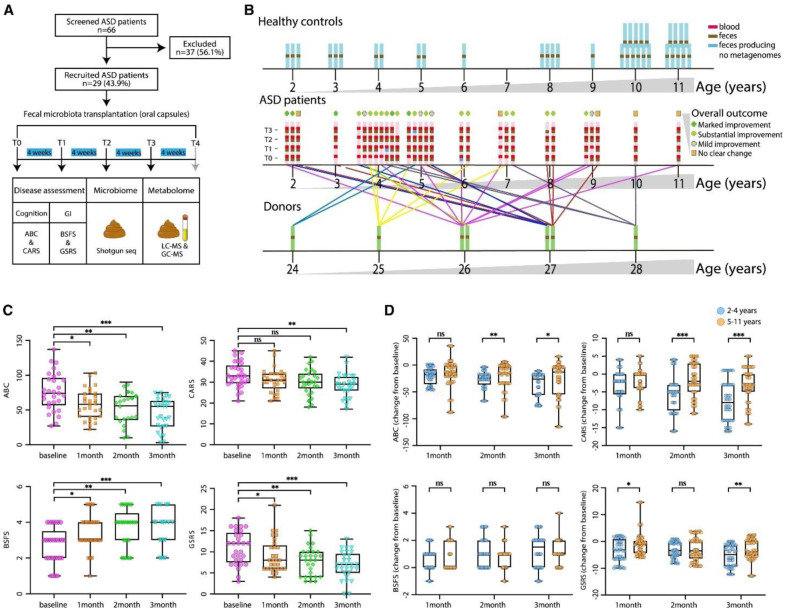
Study design and comprehensive assessment of cognitive and gastrointestinal functions in children with ASD undergoing fecal microbiota transplantation (FMT). (adapted from [186] under the CC-BY 4.0 license).

Chen et al. conducted an open-label study on 29 children with ASD and gastrointestinal (GI) symptoms and administered four monthly sessions of oral fecal microbiota transplantation (FMT) capsules without antibiotic pretreatment. They assessed cognitive and GI functions via the Autism Behavior Checklist (ABC), Childhood Autism Rating Scale (CARS), Bristol Stool Form Scale (BSFS), and Gastrointestinal Symptom Rating Scale (GSRS) at baseline and at multiple points during the 4-month treatment (Figure 9). The study revealed significant gradual improvements in both cognitive symptoms and GI functions, with younger children exhibiting greater benefits, demonstrating the potential of FMT to improve core ASD symptoms alongside GI comorbidities through gut microbiota modulation [186].

A critical analysis revealed that while the associations among gut microbiome dysbiosis, altered neurotransmitter function, and ASD are well established, the exact causal mechanisms and pathways remain areas of active research. The multifactorial etiology of ASD, involving genetic predispositions, environmental stressors, and immune dysregulation, further complicates the understanding of the precise contribution of the gut microbiome [187]. Future research needs to employ larger, longitudinal studies with robust methodologies to standardize findings across populations and to thoroughly investigate the functional consequences of specific microbial alterations on neurotransmitter synthesis, release, and receptor sensitivity. This includes detailed mechanistic studies, perhaps utilizing advanced omics technologies to profile microbial metabolites and host responses, to pinpoint specific microbial–host interactions that drive changes in neurochemistry. Understanding the individual variability in microbiota composition and its impact on neurochemical pathways will be crucial for developing personalized and effective microbiome-targeted therapies for ASD [188].

## 8. Challenges and future directions

**Figure 10.**
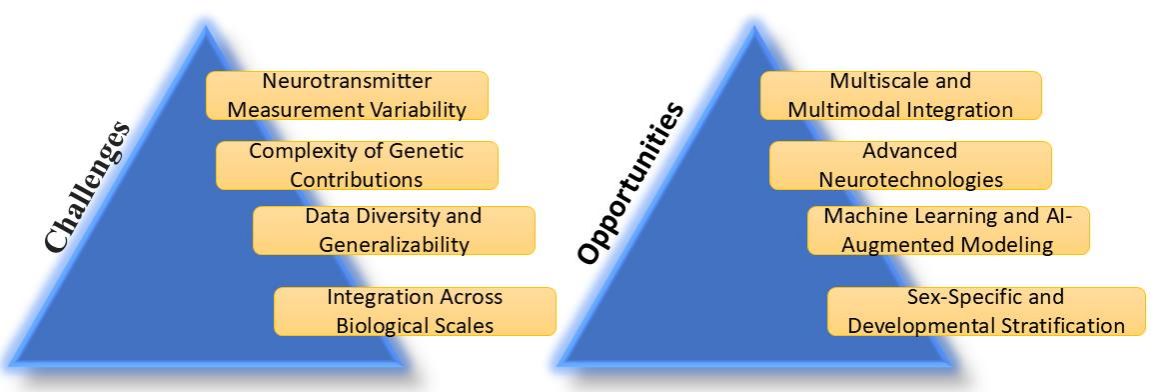
Key challenges and opportunities in computational modeling of the E/I balance in individuals with ASD.

The E/I balance model aimed at screening for ASD is a subject of several problems, such as data heterogeneity, the complexity of nature, and a poor translational pipeline (Figure 10) [189]. Although sophisticated simulation systems, including NEURON, NEST, and the virtual brain, operate, neurotransmitter dynamics and genetic variations are highly integrated. Indicator GABAergic and glutamatergic signaling are frequently simplified by models and do not overwhelm the alterations that are possible across developmental stages or subtypes of ASD [190]. The inconsistency observed among individuals, across brain regions, and at various time points presents substantial hurdles in the development of universally applicable models [191]. Moreover, every region and age group varies, which complicates the standardization of biomarkers in magnetic resonance spectroscopy results [192].

Genetic data also introduce layers of complexity. Neurotransmitter pathways are influenced by polygenic interactions, rare mutations and epigenetic changes, which are not easily captured within the framework of the current models. In silico models are frequently centered around a small group of genes, e.g., SHANK3 or GABRB3, and overlook the greater genetic configuration involving ASD. Additionally, there is a barrier to converting model predictions to clinical practice. The estimation of the E/I balance remains an area of personalized medicine, and noninvasive, real-time monitoring devices in use present a limitation to such estimation efforts; clinical use also poses regulatory and ethical issues. The biophysically realistic simulation and the absence of cross-species validation also delay clinical translation because of the computational cost needed.

Despite these challenges, the field offers considerable opportunities. Multiscale computational models that combine genetics, neurotransmitter dynamics, and brain connectivity are emerging as powerful tools for understanding ASD heterogeneity. These models can stratify patient subtypes on the basis of molecular and electrophysiological signatures, potentially guiding precision medicine strategies. Machine learning-enhanced simulations can process large-scale multiomics data to refine E/I balance estimations, improving diagnostic capabilities and early detection [60].

Neurotechnology advancements, including EEG-based functional E/I estimation and fMRI-informed connectomics, open new possibilities for individualized therapeutic modeling. Novel therapeutic strategies, such as combining pharmacologically modulated E/I balance with transcranial stimulation techniques, hold promise for targeted interventions. Integrating environmental factors, microbiome interactions, and sex-specific differences into computational pipelines can further refine these models.

Prospects include the development of real-time E/I balance monitoring tools, cross-platform open-source modeling initiatives, and collaborations between computational neuroscientists, clinicians, and geneticists. Future directions should also focus on making simulations scalable and cost-effective, with an emphasis on longitudinal, developmentally informed models that bridge the gap between bench and bedside. The integration of computational E/I balance modeling into clinical trials, alongside pharmacogenomics and neuroimaging, represents a promising path toward personalized ASD interventions.

## 9. Conclusion

Computational modeling of E/I balance is a rapidly evolving interdisciplinary field crucial for unraveling the neurobiology of ASD. In this review, we synthesize the complex interplay between glutamatergic and GABAergic neurotransmitter systems and genetic factors, including mutations in key genes such as *SHANK3*, *GRIN2A*, and *GABRB3*, which collectively underline ASD pathophysiology. Advanced computational tools have yielded critical insights by linking molecular and synaptic disruptions to circuit-level dysfunctions and behavioral phenotypes. A conceptual framework illustrates how genetic mutations disrupt synaptic proteins and receptor functions, producing an imbalance in glutamatergic and GABAergic signaling that propagates through neural networks. This dysregulated E/I ratio manifests as aberrant oscillations and impaired information processing, correlating with core behavioral symptoms of ASD such as social communication deficits and repetitive behaviors. Computational models are integrative platforms to simulate these multilevel alterations, enabling in silico testing of hypotheses that inform personalized therapeutic interventions. By simulating patient-specific molecular and electrophysiological profiles, such models hold promises for stratifying ASD heterogeneity, predicting treatment response, and identifying novel targets aimed at restoring E/I balance. Nonetheless, challenges remain, including inconsistencies in neurotransmitter measurements, polygenic complexity, and the pressing need for cross-species and longitudinal validation. The future of ASD research lies in the synergy of multiomics data integration, machine learning, and scalable multiscale modeling. Developing comprehensive models incorporating genetic, neurochemical, and environmental factors will enable translational advances, turning computational predictions into clinical tools that improve diagnosis, intervention, and long-term management of autism.

## Use of AI tools declaration

The authors declare they have not used Artificial Intelligence (AI) tools in the creation of this article.
